# In-hospital mortality for aspiration pneumonia in a tertiary teaching hospital: A retrospective cohort review from 2008 to 2018

**DOI:** 10.1186/s40463-022-00617-2

**Published:** 2023-03-07

**Authors:** Dongho Shin, Gerald Lebovic, R. Jun Lin

**Affiliations:** 1 Department of Otolaryngology – Head and Neck Surgery, University of Toronto, Toronto, Canada; 2 Biostatistics (Canada) Global Database Studies, IQVIA, Mississauga, Canada; 3 Department of Otolaryngology – Head and Neck Surgery, University of Toronto, Toronto, Canada

**Keywords:** Aspiration pneumonia, Oropharyngeal dysphagia, In-hospital mortality

## Abstract

**Background:**

Aspiration pneumonia is a preventable condition that has higher rates of recurrence and mortality compared to non-aspiration pneumonia. The primary objective of the study was to examine independent patient factors that are associated with mortality in those requiring acute admission for aspiration pneumonia at a tertiary institution. Secondary goals of the study were to review whether factors such as mechanical ventilation and speech language pathology intervention can impact patient mortality, length of stay (LOS), and costs relating to hospitalization.

**Methods:**

Patients older than 18 years of age who were admitted with a primary diagnosis of aspiration pneumonia from January 1, 2008 to December 31, 2018 at Unity Health Toronto-St. Michael’s hospital in Toronto, Canada, were included in the study. Descriptive analyses were performed on patient characteristics using age as a continuous variable as well as a dichotomous variable with age 65 as a cut-off. Multivariable logistic regression was used to identify independent factors that contributed to in-hospital mortality and Cox proportional-hazard regression was used to identify independent factors that affected LOS.

**Results:**

A total of 634 patients were included in this study. 134 (21.1%) patients died during hospitalization with an average age of 80.3 ± 13.4. The in-hospital mortality did not change significantly over the ten-year period (*p* = 0.718). Patients who died had longer LOS with a median length of 10.5 days (*p* = 0.012). Age [Odds Ratio (OR) 1.72, 95% Confidence Interval (95% CI) 1.47–2.02, *p* < 0.05] and invasive mechanical ventilation (OR 2.57, 95% CI 1.54–4.31, *p* < 0.05) were independent predictors of mortality while female gender was found to be a protective factor (OR 0.60, 95% CI 0.38–0.92, *p* = 0.02). Elderly patients had five times higher risk of dying during their hospital course when compared to younger patients [Hazard Ratio (HR) 5.25, 95% CI 2.99–9.23, *p* < 0.05).

**Conclusion:**

Elderly patients are a high-risk population for developing aspiration pneumonia and are at higher risk of death when hospitalized for this condition. This warrants improved preventative strategies in the community. Further studies involving other institutions and creating a Canada-wide database are required.

**Graphical Abstract:**

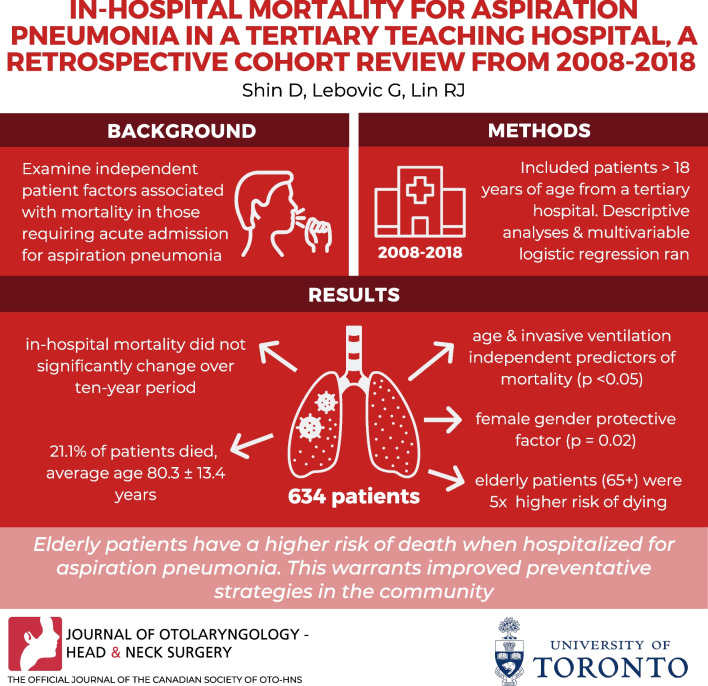

## Introduction

Aspiration pneumonia is an infectious pulmonary process that is caused by inhalation of oropharyngeal secretions or gastric contents colonized by pathogenic bacteria into the lower respiratory tracts [1]. It accounts for approximately 5–15% of all community-acquired pneumonias, which equated to an incidence of 7.1 cases per 10,000 people in the United States in 2012 [2]. Patients with multiple risk factors including advanced age, those who have suffered from cerebrovascular accidents, underlying neurological conditions, or previous major head and neck surgery and/or radiation treatments have increased rates of aspiration pneumonia [3–11]. A meta-analysis showed that frail elderly patients with dysphagia have about 9 times higher odds of developing aspiration pneumonia and this odds increased to 13 times higher if they have concurrent cerebrovascular disease [12]. Such patients were seen to have worse clinical outcomes resulting in prolonged hospitalization, particularly in frail, elderly patients with a mortality rate as high as 10% [2, 12].

Management of these patients often involve positioning of patients to prevent further aspiration, with frequent suctioning of oropharyngeal contents [1]. Antibiotics are often initiated and bronchoalveolar lavage can help to direct antibiotic choice [1]. Patients are also administered oxygen with close oxygen saturation monitoring. In those with signs of hypoxia, intubation and mechanical ventilation may be warranted, requiring intensive care unit (ICU) admission [1]. Although accurate data on incidence of aspiration pneumonia requiring ICU admission is not currently available, Leroy et al*.* saw 23% of patients admitted to ICU for severe community-acquired pneumonia to be caused by aspiration pneumonia [13]. Even with ICU level of care, complications can arise including acute respiratory distress syndrome, empyema, abscess, and respiratory failure. In addition to mortality rates being high, recovery can also be a prolonged process with repeated admissions [1].

Despite these facts, institution-level and population-level data evaluating patients admitted for aspiration pneumonia in Canada is severely limited [14–16]. Unity Health Toronto-St. Michael’s Hospital in Toronto, Ontario, Canada, is a tertiary teaching and research center that caters to approximately 6 million people in the metro Toronto region and therefore is ideal in collecting large institutional data on this patient population. The primary goal of the current study is to examine independent patient factors that are associated with mortality in those requiring acute admission for aspiration pneumonia at St. Michael’s hospital from 2008 to 2018. Secondary goals of the study include investigation of whether medical interventions such as mechanical ventilation and speech language pathology (SLP) treatment can impact patient mortality, LOS, as well as direct and indirect costs relating to hospitalization.

## Methodology

This study was approved by Unity Health Toronto Research Ethics Board (REB #20-226). This retrospective cohort study was performed at a single tertiary teaching institution in Toronto, Ontario. Inclusion criteria included patients older than 18 years of age who were admitted at St. Michael’s Hospital, with a primary diagnosis of aspiration pneumonia from January 1, 2008 to December 31, 2018. Patients younger than the age of 18, those with secondary diagnosis of aspiration pneumonia (those who were already hospitalized, and subsequently developed pneumonia as a secondary complication) were excluded from this study. Decision Support Services at St. Michael’s Hospital helped identify patients who fit our inclusion and exclusion criteria based on International Classification of Diseases-10 (ICD-10). ICD-10 code J69.0 (pneumonitis due to inhalation of food and vomit) was used to define aspiration pneumonia. Decision Support Services is a team of analysts who provides analytical support in data management and health information to healthcare programs across Unity Health Toronto to support evidence informed decision making, as well as performance monitoring and improvement.

Patient baseline characteristics including age, gender, date of admission and discharge, and number of medical comorbidities (categorized into 0, 1, ≥ 2) were collected. Charlson comorbidity index (CCI) was used to calculate patients’ comorbidities to predict in-hospital mortality [17, 18]. In-hospital mortality was the primary study outcome while patients’ LOS, need for invasive mechanical ventilation, speech language pathology (SLP) involvement, and hospitalization costs were secondary outcomes. Hospitalization costs were divided into [1] direct costs, which were calculated by the dollar expenses relating to direct patient care such as nursing, laboratory expenses, medications, etc.; and [2] indirect costs were calculated as the hospital overhead costs from areas that do not provide patient care, but support the infrastructure of the organization such as information technology, engineering, health records, and administration. Adjustments for inflation were not taken into consideration in this study.

Statistical analyses were performed using R version 4.0.2 (R Core Team, Vienna, Austria). Descriptive analysis compared groups of interest using parametric or non-parametric t-tests for continuous variables as appropriate and Chi-Square tests for categorical variables. We compared those who died from aspiration pneumonia versus those who did not, as well as those who were age 65 and older (≥ 65) versus those who were under the age 65. We used a multivariable logistic regression model for the mortality outcome and partial residuals were used to assess the functional form of the continuous variables. For the LOS outcome, a Cox-Proportional Hazards model as well as cumulative incidence curves were used due to the competing risk of death. The primary analysis used age as a continuous variable and a secondary analysis dichotomized age using a cut-off of 65. Although dichotomizing continuous variables can reduce power, this was done as people older than age 65 are typically considered to be geriatric for healthcare. Variables assessed in the regression models included age, year from study start, CCI, gender, invasive mechanical ventilation, and SLP involvement. A two-sided *p*-value of < 0.05 was used as the criterion for statistical significance. Model validation and calibration was performed using 100 bootstrap samples [19].

## Results

A total of 634 patients who were admitted with a primary diagnosis of aspiration pneumonia from January 1, 2008 to December 31, 2018 were identified by the Decision Support Services and included in the study. No patients were excluded from the list provided by the Decision Support Services. Of these patients, 134 (21.1%) had in-hospital mortality and they were significantly older compared to the group that survived (age 80.3 ± 13.4 vs. 68.2 ± 19.3 respectively, *p* < 0.001) (Table 1). A linear relationship between increasing age and mortality was found. Patients who died had longer LOS with a median length of 10.5 days (*p* = 0.012). Although there were no significant gender differences between the two groups (*p* = 0.20), patients who died during hospitalization had higher CCI scores (*p* = 0.01) and were more likely to be mechanically ventilated (*p* = 0.007) (Table 1). Approximately 44% of patients received SLP intervention during their hospitalization and this was comparable between the two groups. Overall, the admission rate for aspiration pneumonia was relatively stable over the 10-year study period.

**Table 1:** Summary statistics of included patients based on in-hospital mortality

Clinical parameters	In-hospital mortality		
	No (n = 500)	Yes (n = 134)	*p*-value
Age, mean (SD)	68.2 (19.3)	80.3 (13.4)	< 0.001
Gender, male (%)	311 (62.2)	92 (68.7)	0.201
Year of admission, n (%)			0.718
2008	39 (7.8)	10 (7.5)	
2009	47 (9.4)	15 (11.2)	
2010	51 (10.2)	18 (13.4)	
2011	31 (6.2)	5 (3.7)	
2012	49 (9.8)	10 (7.5)	
2013	52 (10.4)	7 (5.2)	
2014	48 (9.6)	14 (10.4)	
2015	47 (9.4)	14 (10.4)	
2016	45 (9.0)	14 (10.4)	
2017	52 (10.4)	14 (10.4)	
2018	39 (7.8)	13 (9.7)	
Number of comorbidities, n (%)			0.041
0	1 (0.2)	0 (0.0)	
1	79 (15.8)	10 (7.5)	
≥ 2	420 (84.0)	124 (92.5)	
CCI, median (IQR)	1.0 (0.0, 2.0)	2.0 (1.0, 3.0)	0.011
Invasive mechanical ventilation, n (%)	72 (14.4)	33 (24.6)	0.007
Total LOS, median (IQR)	6.0 (3.0, 13.0)	10.5 (3.3, 19.0)	0.012
Total direct cost, median $ (IQR)	6208.7 (3142.0, 12,962.4)	10,161.2 (3756.7, 26,627.3)	0.002
Total indirect cost, median $ (IQR)	2249.5 (1073.0, 4453.2)	3594.7 (1284.3, 8833.3)	0.005
SLP intervention, n (%)	223 (44.6)	59 (44.0)	0.984

*SD* Standard deviation, *CCI* Charlson comorbidity index, *IQR* interquartile range, *LOS* Length of stay, *SLP* Speech language pathology

Dichotomizing using an age cut-off of 65, there were 202 (31.9%) patients who were younger than 65 years of age with a mean age of 47.4 ± 12.8 and 432 (68.1%) patients who were 65 or older, with a mean age of 81.7 ± 8.4 (Table 2). In-hospital mortality rate was significantly higher in the older age group (*p* < 0.001) and older patients had higher numbers of medical comorbidities (*p* < 0.001) and higher CCI scores (*p* < 0.001). Interestingly, older patients had similar LOS (6.0 vs. 6.5 days, *p* = 0.612) and had lower rates of mechanical ventilation (14.4% vs. 21.3%, *p* = 0.038) when compared to the younger group (Table 2).

**Table 2:** Summary statistics dichotomized by age

Clinical parameters	Age		
	< 65 (n = 202)	≥ 65 (n = 432)	*p*-value
Age, mean (SD)	47.4 (12.8)	81.7 (8.5)	< 0.001
Gender, male (%)	145 (71.8)	258 (59.7)	0.004
In-hospital mortality, n (%)	14 (6.9)	120 (27.8)	< 0.001
Number of comorbidities, n (%)			0.011
0	1 (0.5)	0 (0.0)	
1	39 (19.3)	50 (11.6)	
≥ 2	162 (80.2)	382 (88.4)	
CCI, median (IQR)	1.0 (0.0, 2.0)	2.0 (1.0, 3.0)	< 0.001
Invasive mechanical ventilation, n (%)	43 (21.3)	62 (14.4)	0.038
Total LOS, median (IQR)	6.5 (3.0, 15.8)	6.0 (3.0, 14.0)	0.612
Total direct cost, median $ (IQR)	7908.9 (3515.9, 19,558.7)	6603.4 (3131.47, 13,745.2)	0.101
Total indirect cost, median $ (IQR)	2682.2 (1184.9, 6223.5)	2328.9 (1048.8, 4663.4)	0.072
SLP intervention, n (%)	66 (32.7)	216 (50.0)	< 0.001

*SD* Standard deviation, *CCI* Charlson comorbidity index, *IQR* interquartile range, *LOS* Length of stay, *SLP* Speech language pathology

Multivariable logistic regression analyses were performed first using age as a continuous variable then as a dichotomized variable with age 65 as a cut-off. With age as a continuous variable, age per 10-year increase and mechanical ventilation were found to be independent predictors of in-hospital mortality (OR 1.72, 95% CI 1.47–2.02, *p* < 0.05 and OR 2.57, 95% CI 1.54–4.31, *p* < 0.05, respectively), whereas female gender was found to be protective against in-hospital mortality (OR 0.60, 95% CI 0.38–0.92, *p* = 0.02) (Table 3). SLP intervention appeared to be protective against in-hospital mortality but did not reach statistical significance. With age as a dichotomized variable, again age 65 and above and mechanical ventilation were found to be independent predictors of in-hospital mortality (OR 6.11, 95% CI 3.33–11.23, *p* < 0.05, and OR 2.34, 95% CI 1.42–3.88, *p* < 0.05, respectively) (Table 3). Female gender was no longer protective against in-hospital mortality in this model (OR 0.67, 95% CI 0.44–1.03, *p* = 0.07), neither was SLP intervention.

**Table 3:** Multivariable logistic regression for in-hospital mortality

	OR	Confidence interval (95%)	*p*-value
*Age as continuous variable*			
Age per 10 year increase	1.72	1.47–2.02	< 0.001
CCI	1.04	0.94–1.16	0.43
Year from study start	1.01	0.95–1.08	0.72
Gender (F:M)	0.60	0.38–0.92	0.02
Invasive mechanical ventilation (Yes: No)	2.57	1.54–4.31	< 0.001
SLP consult (Yes:No)	0.69	0.46–1.05	0.09
*Age dichotomized*			
Age (≥ 65: < 65)	6.11	3.33–11.23	< 0.001
CCI	1.04	0.93–1.15	0.50
Year from study start	1.01	0.95–1.08	0.79
Gender (F:M)	0.67	0.44–1.03	0.07
Invasive mechanical ventilation (Yes: No)	2.34	1.42–3.88	< 0.001
SLP intervention (Yes:No)	0.76	0.51–1.15	0.20

*OR* Odds ratio, *CCI* Charlson comorbidity index, *SLP* Speech language pathology

A Cox proportional-hazard model was used to control for variables including year from study start, CCI, age (≥ 65 vs. < 65), gender, invasive mechanical ventilation and SLP intervention (Table 4). Elderly patients were identified to have approximately five times higher risk [Hazard Ratio (HR) 5.25, 95% CI 2.99–9.23, *p* < 0.05] of dying during their hospital course when compared to younger patients. Cumulative incidence curves showed that of the study sample, 90% of the participants were discharged or died within 120 days.

**Table 4:** Length of stay and mortality as a competing risk

	HR	Confidence interval (95%)	*p*-value
Age (≥ 65: < 65)	5.25	2.99–9.23	< 0.001
CCI	0.97	0.88–1.06	0.48
Year from study start	1.03	0.98–1.09	0.29
Gender (F:M)	0.86	0.59–1.26	0.45
Invasive mechanical ventilation (Yes: No)	0.62	0.41–0.95	0.03
SLP intervention (Yes:No)	0.50	0.35–0.72	< 0.001

*HR* Hazard ratio, *CCI* Charlson comorbidity index, *SLP* Speech language pathology

In general, the total yearly cost from 2008 to 2018 increased from $956′959.60 to $1′287′956.50. Patients who died had significantly higher total direct ($10′161.20 per patient, *p* = 0.002) and indirect costs ($3′594.70 per patient, *p* = 0.005) when compared to those who survived (Table 1). When age was dichotomized to elders and those younger than 65, there was no statistically significant difference between the two groups (Table 2).

Model validation and calibration indicated some minor overfitting. The apparent Somers D_xy_ was 0.40 while the bias corrected was 0.37 which resulted in a minor optimism of 0.03. Calibration in the large had an optimism of 0.12 and calibration of the slope was 0.91 indicating some minor overfitting.

## Discussion

Overall, this large institutional data review found that increasing age and the need for mechanical ventilation during hospitalization were independent risk factors for in-hospital mortality in patients admitted with a primary diagnosis of aspiration pneumonia. Patients older than age 65 were 5 times more likely to die during their admission compared to younger patients. LOS was comparable between the two age groups, most likely due to the higher mortality rate seen in the older patient population.

### Impact of age on in-hospital mortality

**Fig. 1:**
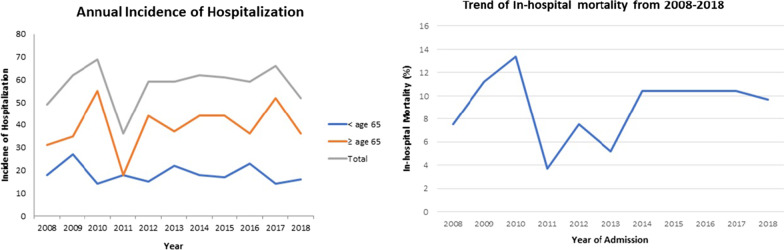
Trend of aspiration pneumonia from 2008 to 2018

Out of the 634 patients admitted for aspiration pneumonia from 2008 to 2018, majority of patients (68.1%) were of age 65 or older, with a mean age of 81.7 ± 8.5. The average age of the patients that died during admission was 80.3 ± 13.4. Over the 10-year study period excluding the year 2011, our data have shown that there were at least 1.5 times more cases of aspiration pneumonia requiring admission in those aged 65 or older compared to the younger patients (Fig. 1). We know from existing evidence that the incidence of aspiration pneumonia is higher in the elderly [2]. As the human body undergoes the natural aging process, cerebral atrophy, decreased nerve function, and muscle atrophy can all drastically impact the swallowing function [20]. A case–control study by Almirall et al*.* showed that oropharyngeal dysphagia significantly increased the risk of pneumonia (OR 11.9) and 92% of patients diagnosed with pneumonia had signs of oropharyngeal dysphagia on video fluroscopic swallowing study (VFSS) [3]. Even in otherwise healthy elders, there are video fluoroscopic and radiological changes in the oral and pharyngeal phases of swallowing when compared to healthy, young adults [21–23]. Further, elders’ predisposition to multiple comorbidities puts them at higher risk of aspiration pneumonia [20]. Medical comorbidities such as pulmonary diseases, stroke, dementia, or Parkinson’s disease can devastatingly affect various mechanical aspects of oropharyngeal swallowing, leading to aspiration [5, 24–28]. This trend was observed in our patient population, where those aged 65 or higher had a higher proportion of individuals with two or more comorbidities (88.4% vs 80.2%, *p* = 0.011) and higher CCI scores (2.0 vs 1.0, *p* < 0.001) compared to the younger group.

### Unexpected findings

When comparing the two dichotomized age groups, elderly patients were less likely to receive mechanical ventilation (14.4 vs. 21.3%; *p* = 0.038), even when mortality rate was higher in this group (27.8%, *p* < 0.001) (Table 2). This was an unexpected finding as one would assume a group associated with higher mortality would receive more aggressive and invasive treatments. Previous studies have shown that other factors such as respiratory physiology, burden of comorbidities, and severity of acute illness all contributed to outcomes in mechanical ventilation in the elderly [32, 33]. Therefore age is often a modifier of the effect of disease and not a sole decision altering factor when determining the usage of mechanical ventilation in this population [29]. Further, many elderly patients likely have specific goals of care and therefore “Do Not Resuscitate” orders in this population can further limit intubation and aggressive treatments.

The study has found that SLP involvement was not protective against in-hospital mortality in our study. This may not be surprising because the patients admitted already had aspiration pneumonia and therefore the underlying issues that had led to aspiration were already present. Additionally, given the relatively short LOS of patients admitted for aspiration pneumonia, the full benefit of swallowing rehabilitation may not have been completely elucidated by the current study.

The data for the current study were captured by Decision Support Services, which is a powerful database collecting patient clinical data for care quality improvement and research purposes. We had no missing data for the current study and our unexpected findings will need to be further explored via a larger, more diverse study population from different hospitals regionally and nationally.

### Public health implications

The current study demonstrated an alarming in-hospital mortality rate of 27.8% in those over age 65 who were hospitalized with a primary diagnosis of aspiration pneumonia. In 2020, 18% (6.8 million) of the Canadian population was 65 years of age or older [30]. The prevalence of undiagnosed dysphagia in community dwelling elders, a major risk factor for aspiration pneumonia, can be up to 15% [31]. This prevalence increases to 31% in those living in nursing homes [32]. With the increasing aging population in Canada, preventative measures against aspiration pneumonia in the elderly population should become a public health priority. Community programs such as dysphagia screening, including baseline SLP screening, SLP education on oral care, diet modifications, nutrition, as well as dysphagia rehabilitation can help decrease admission to hospital for aspiration pneumonia. Such trends were seen in the United States, incidence of aspiration pneumonia in the elderly population decreased from 40.7 to 30.9 cases per 10,000 people from 2002 to 2012 with implementation of public preventative programs [2, 28, 33]. In-hospital mortality also decreased from 18.9 to 9.8% during the same time period due to reduced disease severity and improved delivery of care to the elderly population [2]. This can not only help prevent admission, but also decrease hospital costs, and ultimately death.

### Limitations

There were several limitations to our study. The inclusion criteria were limited to those with a primary, not secondary, diagnosis of aspiration pneumonia. Primary diagnosis was the label given for the main diagnosis for the patients’ hospital admission. Secondary diagnosis was given when patients developed another disease in addition to the primary diagnosis. Study patients were identified by Decision Support Services at St. Michael’s Hospital using diagnostic codes (ICD-10). Code misclassification is possible in administrative database analysis. Additionally, the ICD-10 code used was J69.0, pneumonitis due to inhalation of food and vomit. This code does not distinguished between whether the aspirant was gastric in origin or oropharyngeal in origin, which may have implications as to whether the aspiration can result in bacterial pneumonia versus chemical pneumonitis. In reality, however, it is very difficult to differentiate the quality of the aspirate and often times a combination of chemical and bacterial aspirates occur [34, 35]. Our patient population was also restricted to one institution in Toronto, which can limit the variability of patient characteristics and treatment preferences. However, our large patient population and completeness of data collection helped mitigate these limitations and our sample size had ample power for analysis. Future work including multiple institutions, patients with secondary diagnosis of aspiration pneumonia while in hospital, as well as incorporating more patient and treatment characteristics will further identify other independent risk factors for treatment outcomes of aspiration pneumonia. A nation-wide database will strengthen the call for dysphagia screening program to prevent aspiration pneumonia and death, particularly in the elderly population. 

## Conclusion

This study demonstrates elderly patients as a high-risk population for aspiration pneumonia and are at higher risk of death when hospitalized for this condition, warranting improved preventative strategies in the community. Further studies involving other institutions and creating a Canada-wide database are required.

## Data Availability

The datasets used and/or analysed during the current study are available from the corresponding author on reasonable request.
